# Major facilitator superfamily efflux pumps in human pathogens: Role in multidrug resistance and beyond

**DOI:** 10.1016/j.crmicr.2024.100248

**Published:** 2024-06-08

**Authors:** Manjusha Lekshmi, Anely Ortiz-Alegria, Sanath Kumar, Manuel F. Varela

**Affiliations:** a Department of Biology, Eastern New Mexico University, Portales, NM 88130, United States; b QC Laboratory, Post Harvest Technology, ICAR-Central Institute of Fisheries Education (CIFE), Mumbai 400061, India

**Keywords:** Bacteria, Pathogen, Major facilitator superfamily, Antimicrobial resistance, Multidrug resistance, Multidrug efflux pumps

## Abstract

The major facilitator superfamily (MFS) of proteins constitutes a large group of related solute transporters found across all known living taxa of organisms. The transporters of the MFS contain an extremely diverse array of substrates, including ions, molecules of intermediary metabolism, and structurally different antimicrobial agents. First discovered over 30 years ago, the MFS represents an important collection of integral membrane transporters. Bacterial microorganisms expressing multidrug efflux pumps belonging to the MFS are considered serious pathogens, accounting for alarming morbidity and mortality numbers annually. This review article considers recent advances in the structure-function relationships, the transport mechanism, and modulation of MFS multidrug efflux pumps within the context of drug resistance mechanisms of bacterial pathogens of public health concerns.

## Highlights

• The major facilitator superfamily contains solute transporters that are conserved across all living taxa.
• Bacterial pathogens harbor multidrug efflux pumps that compromise the clinical treatment of infection.
• Serious bacterial pathogens harbor multiple antimicrobial resistance mechanisms.
• Multidrug efflux pumps of the major facilitator superfamily share a common mechanism of antimicrobial transport.
• Antimicrobial efflux pumps represent desirable targets for modulation.

## Graphical abstract

**Figure:**
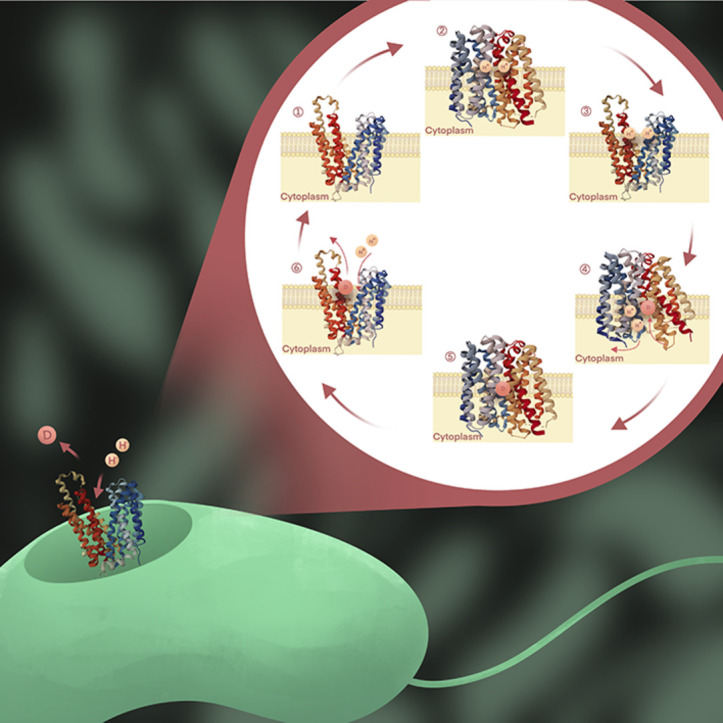

## 1 Introduction

The major bacterial pathogens responsible for human morbidity and mortality belong to Gram-positive and -negative groups, each capable of causing a wide range of infections from skin infections and gastroenteritis to fatal septicaemia or meningitis. The era of antimicrobial treatment that penicillin heralded in the 20^th^ century continues today and has largely been able to control infectious diseases of various forms. However, using antibiotics facilitated selection for bacteria that could resist them by various physiological means, followed by the spread of genetic traits of resistance, resulting in emerging strains that could resist multiple antibiotics, termed multidrug-resistant (MDR) bacteria. The World Health Organization (WHO) has identified infections caused by MDR bacteria as the most severe public health crisis that would negatively impact the global healthcare system and lead to large-scale morbidities, mortalities, and consequent economic losses unless immediate measures are taken (O’Neill, 2014; WHO, 2023). MDR bacteria responsible for a majority of infections belong to *Escherichia coli, Staphylococcus aureus, Klebsiella pneumoniae, Streptococcus pneumoniae, Acinetobacter baumannii*, and *Pseudomonas aeruginosa*, being directly responsible for over an estimated 1.27 million deaths in 2019, and associated with over 4.5 million deaths (Murray et al., 2022). *E. coli* is a versatile pathogen capable of causing intestinal and extraintestinal infections. Various host ranges, including humans, animals, and birds, are sources of pathogenic *E. coli* that enter the food chain through fecal contamination. Members of the diarrheagenic *E. coli* are grouped into enterotoxigenic (ETEC), enteropathogenic (EPEC), enterohemorrhagic/Shiga toxin-producing (EHEC/STEC), enteroaggregative (EAEC), diffusely adherent (DEAC) and enteroinvasive (EIEC) pathotypes based on the nature of the infection, virulence factors and the serotypes involved (Fratamico et al., 2016; Kaper et al., 2004). The pathogenic *E. coli* have acquired an array of virulence factors, which include enterotoxins, adherence factors, and secretion systems through horizontal gene transfer events, and each of the pathotypes mentioned above harbors a unique combination of the virulence factors that are responsible for the nature of diarrheal disease caused by that particular pathogroup (Gomes et al., 2016; Kaper et al., 2004). A phylogenetically distinct group of *E. coli* known as the extraintestinal pathogenic *E. coli* (ExPEC) causes adult bacteremia, pneumonia, and meningitis in neonates (Russo and Johnson, 2000; Yun et al., 2023). ExPEC are the most common agents of urinary tract infections (Russo and Johnson, 2000). Among Gram-positive bacteria, methicillin-resistant *Staphylococcus aureus* (MRSA), vancomycin-resistant *Enterococcus faecium* (VRE), and MDR *Streptococcus pneumoniae* have become serious concerns both in community and hospital settings (Koulenti et al., 2019). An array of infections caused by these bacteria consist of skin and soft tissue, pulmonary, implant-associated infections, meningitis, sinusitis, bronchitis, pneumonia, and endocarditis (Jubeh et al., 2020). The MRSA strains are variously classified as health-care-associated MRSA (HA-MRSA), community-associated MRSA (CA-MRSA), and livestock-associated MRSA based on the sources of strains, spread of infection, and antimicrobial resistance profiles (Shoaib et al., 2023; Turner et al., 2019).

The Gram-negative bacterial pathogens identified as global priority pathogens by the WHO owing to their extensive resistance to multiple antibiotics include cephalosporin and carbapenem-resistant Enterobacterales (CRE), carbapenem-resistant *Pseudomonas aeruginosa*, and carbapenem-resistant *Acinetobacter baumannii.* Of particular importance are healthcare-associated infection**s** such as ventilator-associated pneumonia, catheter-associated urinary tract infections**,** and bloodstream infections (Kaye and Pogue, 2015; Martirosov and Lodise, 2016). *E. coli, Enterobacter* spp.**,** and *Klebsiella pneumoniae* are the important MDR Enterobacterales responsible for frequent nosocomial infections. Among non-fermenting Gram-negative bacteria, *Pseudomonas aeruginosa* and *Acinetobacter baumannii* are serious pathogens causing difficult-to-treat pneumonia and bloodstream infections due to their extreme drug**-**resistance traits (Kaye and Pogue, 2015). Three prominent agents of nosocomial infections are *E. coli, K. pneumoniae,* and *P. aeruginosa,* which contribute to 27% of all documented infections and 70% of all Gram-negative infections associated with healthcare settings (Kaye and Pogue, 2015; Sievert et al., 2013). Infections with these bacteria occur in both hospital and community settings, and their recalcitrance to currently available antimicrobial treatment results in increased mortality and morbidity in the form of extended hospital stays and high treatment costs.

## 2 Bacterial antimicrobial resistance mechanisms

The bacterial strategies to fend off antibiotics are common across the bacterial community. However, multiple mechanisms may be employed to bring about the same end effect of recalcitrance to a particular antibiotic. These strategies are i) hydrolytic destruction of antibiotics into simpler and ineffective substances, ii) chemically modifying antibiotics such that the modified compounds are unable to perform their original intended tasks, iii) modifying the cellular targets of antibiotics so that the antibiotics are unable to bind and disturb normal physiology of bacteria iii) preventing the entry of antibiotics by closing or modifying their portals of entry on the cell surfaces iv) rapidly pump out antibiotics from the cell so that they do not reach lethal concentrations (Blair et al., 2015; Kumar and Varela, 2013). The hydrolytic destruction of antibiotics is a common and the most effective means of resistance in which antibiotics serve as substrates to one or more enzymes produced by bacteria. The β-lactamases that cleave the β-lactam ring of the penicillin group of antibiotics are widespread in both Gram-positive and -negative groups and include the penicillins, cephalosporins, carbapenems, and monobactams (Varela et al., 2021; G.D. Wright, 2005). Over 1000 β-lactamases identified so far have been classified into four groups, A, C, D, and B, based on their amino acid sequence homologies (Ambler scheme), while the functional classification scheme (Bush-Jacobi-Medeiros scheme) places them in groups 1 to 3 (Bush, 2018; Cantón, 2008; Hall and Barlow, 2005; Sawa et al., 2020). Depending on the substrate specificity, range, and susceptibility to β-lactamase inhibitors, these enzymes are called penicillinases, cephalosporinases, extended-spectrum β-lactamases (ESBLs), and carbapenemases. The early lactamases, penicillinases, were followed by the appearance of plasmid-borne TEM and SHV lactamases with broader substrate ranges that included semisynthetic β-lactams, such as ampicillin and amoxicillin, and subsequent point mutations in these enzymes led to the evolution of extended spectrum-β-lactamases that could hydrolyze synthetic β-lactams of the cephalosporin and monobactam groups, but not the cephamycins and carbapenems (Paterson and Bonomo, 2005; Sirot et al., 1988; Varela et al., 2021).

On the other hand, carbapenemases are much more diverse and versatile, able to hydrolyze penicillins, cephalosporins, monobactams, and carbapenems (Queenan and Bush, 2007). Since the genes encoding resistance enzymes reside on mobile genetic elements such as plasmids and transposons, the rapid dissemination of such genetic elements has resulted in the widespread acquisition of resistance to multiple β-lactam antibiotics, particularly in Enterobacterales, *Pseudomonas aeruginosa, Acinetobacter baumannii*, and others. In particular, carbapenem-resistant Enterobacterales (CRE) offer formidable treatment challenges as they can resist most clinically relevant antimicrobial agents, leaving very limited treatment options.

The second group of enzymes that modify antibiotics into ineffective substances does so by enzymatically adding groups (group transfer) to antibiotics through phosphorylation, glycosylation, nucleotidylation, ribosylation, and acyl and thiol transfer (Varela et al., 2021; Gerard D. Wright, 2005). Chloramphenicol, aminoglycosides, rifamycins, macrolides, and streptogramins are susceptible to inactivation by group transfer mechanisms. Examples of these types of resistance mechanisms include the aminoglycoside modifying enzymes (AME) such as *N*-acetyltransferases (AAC), *O*-adenyltransferases (ANT), and *O*-phosphotransferases (APH) which modify the target aminoglycoside antibiotics by their respective group transfer activities, and the resultant modified antibiotics fail to bind to their target, the 30S ribosomal subunit, allowing bacteria to overcome the inhibitory activity of these antibiotics (Garneau-Tsodikova and Labby, 2016). Similarly, acetylation of chloramphenicol by the antibiotic-modifying enzyme chloramphenicol acetyltransferase (CAT) prevents antibiotic-mediated inhibition of protein synthesis as the modified antibiotic is unable to bind to the 50S ribosomal subunit (Schwarz et al., 2004)

The modification of antimicrobial agent targets subverts the efficacy of the antibiotics as they cannot bind to their preferred cellular targets due to structural changes. A classic example of this resistance mechanism involves DNA gyrase/topoisomerases, which target the fluoroquinolone group of antimicrobial agents. Point mutations in genes encoding these enzymes, *gyrA, gyrB, parC,* and *parE*, result in structural changes in these target proteins that affect drug binding (Aldred et al., 2014; Blair et al., 2015). Similarly, bacteria resist the macrolide, lincosamide, and streptogramin B group of antibiotics by interfering with their target site, the 50S ribosomal subunit, by a post-transcriptional modification of the 23S ribosomal RNA involving the methylation of a key adenine residue (Leclercq, 2002). In Gram-positive bacteria, the penicillin-binding protein (PBP) functions to cross-link amino acids to form peptide chains during peptidoglycan cell wall synthesis. Binding of PBP with β-lactam antibiotics inhibits cell wall formation, resulting in the lysis and death of the bacterium. However, a modified form of PBP known as PBP2a encoded by the *mecA* gene has weak affinity for penicillin/ β-lactam antimicrobials and thus confers resistance against them (Georgopapadakou, 1993)

Antibiotics enter bacterial cells via uptake by cellular receptors or diffusion through porins in the bacterial outer cell membrane (Nikaido, 2003; Pages et al., 2008). Structurally, the porins are monomeric, dimeric, or trimeric proteins, and they can be specific or non-specific regarding the antimicrobial substrates permitted to diffuse through them (Sugawara and Nikaido, 2012). Porins such as the OmpA protein found in the outer membrane of Gram-negative bacteria allow non-specific, passive diffusion of diverse hydrophilic compounds, including antimicrobials such as β-lactams and fluoroquinolones, and bacteria can impede the entry of antimicrobials by changing the porin structure or reducing the number of porins expressed by down-regulating porin-encoding genes (Kapoor et al., 2017; Prajapati et al., 2021).

While most of the above-described resistance mechanisms exhibit a certain degree of substrate specificity, a group of transmembrane proteins termed efflux pumps widely distributed in both Gram-positive and -negative bacteria extrude diverse antimicrobials encompassing dyes, antiseptics, disinfectants, heavy metals as well as antimicrobial agents (Nguyen et al., 2023; Varela, 2019). These integral membrane protein transporters extrude antimicrobials from the inside of the cell to the outside, thereby effectively reducing the antimicrobial concentration to sub-lethal levels. Efflux pumps are an integral part of bacterial physiology involving the extrusion of toxic metabolites, transport of Krebs cycle intermediates, and signaling molecules involved in quorum sensing and virulence, and play critical roles in their survival and persistence (Lekshmi et al., 2018; Levy, 2002). Efflux pumps that belong to the “primary active transporter” group derive their energy from the hydrolysis of ATP. In contrast, the “secondary active transporter” groups energize their efflux activity using the electro-potential gradient across the membrane (Davidson et al., 2008; Shi, 2013; West, 1980). The number, types, and activities of efflux pumps vary widely among bacterial species; for example, the *E. coli* genome has 29 putative efflux pumps/drug transport systems, while *P. aeruginosa* has 34 such pumps, the functions of a majority of which are not yet determined (He et al., 2004; Saier et al., 1998).

## 3 Antimicrobial efflux pump systems

Efflux pumps of secondary active transporter type are grouped under four superfamilies based on their amino acid sequence similarity, evolutionary origin, structure, and the energization mode, namely (i) the major facilitator superfamily (MFS), (ii) the resistance-nodulation-cell division (RND) superfamily, (iii) the drug/metabolite transporter (DMT) superfamily, and (iv) the multidrug/oligosaccharidyl- lipid/polysaccharide (MOP) superfamily (Hassan et al., 2018; Hvorup et al., 2003; Kumar et al., 2020a; Saier et al., 1999; Varela, 2019). The RND efflux pumps are widely distributed in Gram-negative bacteria and are tripartite in nature, composed of an inner membrane protein pump linked to an outer membrane factor (OMF) by a periplasmic adaptor protein (PAP) (Nikaido, 2011). These efflux proteins traverse the membrane as 12–14 α-helices in a zig-zag manner. The RND efflux pumps are of clinical significance in Gram-negative bacteria. Some extensively studied RND efflux pumps include AcrAB-TolC of *E. coli*, the MexB-MexA-OprM system of *P. aeruginosa*, and AdeABC of *Acinetobacter baumannii* (Nikaido and Takatsuka, 2009; Venter et al., 2015). In contrast, the small multidrug resistance (SMR) family of efflux pumps, which belong to the DMT superfamily, are small proteins with four transmembrane α-helices (Stephen et al., 2023). The MOP superfamily consists of multidrug and toxic compound extrusion transporters (MATE), which use Na^+^ or *H*^+^ electrochemical gradients to energize their efflux activities (Brown et al., 1999; Kuroda and Tsuchiya, 2009). Examples of MATE efflux pumps include YdhE of *E. coli*, NorM and VmrA of *V. parahaemolyticus*, and VcmA and VcrM of non-O1 *V. cholerae* (Huda et al., 2001; Morita et al., 2000, 1998). A relatively new group, the proteobacterial antimicrobial compound efflux (PACE) family, encompasses efflux proteins increasingly recognized as important in Gram-negative bacteria, particularly the proteobacterial group (Hassan et al., 2018). The *Acinetobacter* chlorhexidine efflux AceI, is a prototype PACE family efflux pump that effluxes biocides and short-chain diamines (Hassan et al., 2019, 2015).

## 4 The multidrug efflux pumps of the MFS

### 4.1 Discovery

The notion that sugar transporters from bacteria and humans were related in terms of amino acid sequences and predicted secondary structures led to the early hypothesis that these proteins shared homology and to their grouping into a large transporter superfamily, now called the major facilitator superfamily (MFS) (Maiden et al., 1987). Shortly afterward, seemingly diverse solute transporters of widely distinctive substrates, like sugars, amino acids, and antimicrobial agents, also showed similarities in sequence and membrane topologies, predicting that proteins of the MFS shared a common transport mechanism across the membrane (Griffith et al., 1992). Early studies on these MFS transporters’ physiological properties and energetics showed they were passive and secondary active transporters (Marger and Saier, 1993). In general, the vast majority of the transporters of the MFS harbor 12 or 14 transmembrane segments (TMS) consisting of α-helices that are connected by inter-TMS loops with the N- and C-termini residing on the cytoplasmic face of the membrane (Marger and Saier, 1993) Fig. 1.

**Fig. 1:**
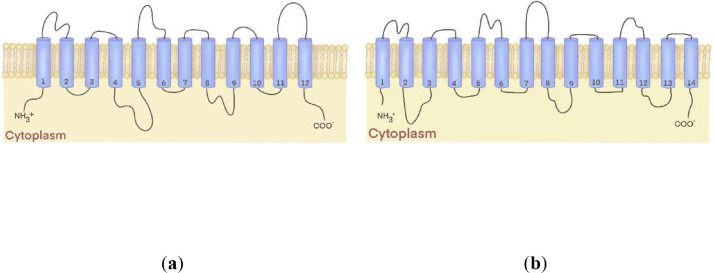
Secondary structures of MFS transporters. The blue cylinders represent membrane-spanning helices, also called transmembrane segments TMSs. MFS transporters are known to harbor 12 TMSs (a) or (b) 14 TMSs across the biological membrane.

The MFS efflux transporter structures harbor 12 or 14 transmembrane segments (TMSs) of α-helical structures connected by cytoplasmic and outward-facing inter-helical, flexible loops. Early studies of MFS transporters focused on analyses of individual amino acid residues and the effects of mutations on the transport activities conferred (Pao et al., 1998). Along these lines, the functional importance of residues belonging to highly conserved signature sequences in various members of the MFS was established (Kumar et al., 2016b). From a more contemporary perspective, the thousands of members belonging to the MFS are systematically placed in a large databank called the Transporter Classification Database (TCD), where sequence and evolutionary relationships are readily accessible (Saier et al., 2021). Some of the extensively studied MFS efflux pumps are those belonging to *S. aureus,* such as QacA, QacB, NorA, NorB, NorC, TetA(K), LmrS, MdeA and MsrA, and Bmr of *Bacillus subtilis* (Costa et al., 2013; Dashtbani-Roozbehani and Brown, 2021; Neyfakh, 1992). EmrD and MdfA of *E. coli* and EmrD-3 of *V. cholerae* are other important antimicrobial efflux pumps of the MFS family (Kumar et al., 2020a).

### 4.2 Importance of the MFS

The MFS efflux transporters are ubiquitous proteins present in all living organisms. Such ubiquity is a known hallmark of one of the largest families of secondary active transporters and one of the oldest, as it has been present for over three billion years (Saier, 1998). Its biological mechanism involves transporting a wide range of substrates by utilizing ion gradients during secondary active transport or substrate gradients during uniport (Drew et al., 2021). The specificity of the substrate can involve a single component; however, MFS proteins can develop a broader spectrum of affinity, allowing bacteria to have additional functions, such as bacterial communication (quorum sensing), protection from the osmotic stress, transport of environmental molecules, biofilm formation, and they can contribute to virulence and offer resistance toward antimicrobial substances (Gaurav et al., 2023; Pasqua et al., 2019). These functional characteristics, the broad spectrum of substrate specificities, and the conservation of sequence and structures suggest that the MFS solute transporters share a common mechanism across all known taxa (Griffith et al., 1992; Pasqua et al., 2019). These shared biological systems allow bacteria better fitness and adaptation to otherwise inhospitable environments (Pasqua et al., 2019).

Furthermore, multidrug resistance (MDR) has been attributed to many MFS efflux pumps by the physiologically observed extrusion of structurally distinct antimicrobial agents (Stephen et al., 2022; Varela et al., 2023). The MFS-based MDR transporters can impose a risk to public health, reduce possibilities for treatment and increase morbidity and mortality from infection (Kim et al., 2021). The functional mechanisms of these MFS antimicrobial transporters remain unclear, given their diverse dynamics. It is imperative to pursue such understanding, as it can permit the development of strategies for designing inhibitors that can work synergistically with known antimicrobials (Yang et al., 2017).

### 4.3 Structure-function relationships

Molecular structural and mechanistic features of the MFS transporters that conferred substrate efflux began to emerge with the advent of X-ray diffraction analyses on crystallized proteins, lending insight upon the comparison of the structures formed by EmrD, MdfA, YajR, all from *E. coli*, LmrP from *Lactococcus lactis*, and NorA from *S. aureus* (Debruycker et al., 2020; Ranaweera et al., 2015), Fig. 2. In general, several features appear to be shared among the multidrug efflux pump systems of the MFS structures. The multidrug efflux pumps of the MFS consist of two global bundles related in terms of amino acid sequence and are composed of the so-called MFS fold (Radestock and Forrest, 2011). The two bundles are thought to be functionally asymmetrical. The N-terminal bundle or domain comprises the TMSs one through six, while the C-terminal domain comprises TMSs 7 through 12.

**Fig. 2:**
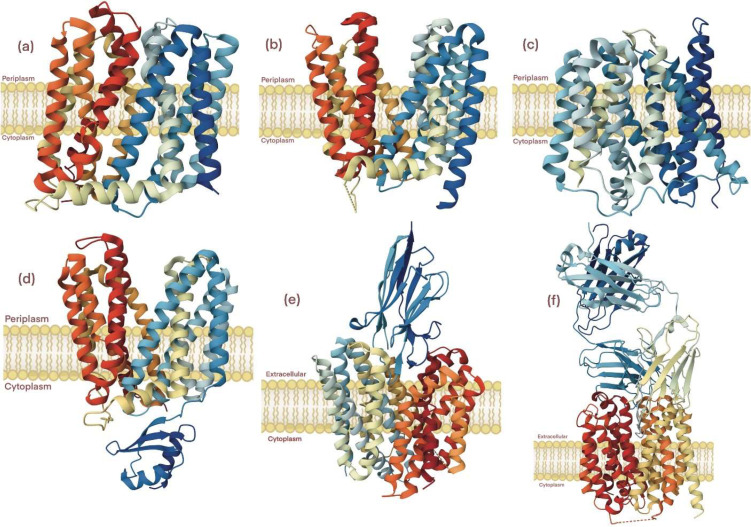
Crystal structures depicting known three-dimensional protein conformations of MFS efflux transporters; (a) MdfA from *E. coli,* (Heng et al., 2015; Liu et al., 2016; Sigal et al., 2007), (b) LmrP from L. *lactis*, (Debruycker et al., 2020), (c) EmrD from *E. coli,* (Baker et al., 2012; Yin et al., 2006), (d) YajR from *E. coli,* (Jiang et al., 2014, 2013), (e) NorA complexed to Fab36, from *S. aureus*, (Brawley et al., 2022), and (f) NorC complexed to ICab3, from *S. aureus*, (Kumar et al., 2020b).

From a molecular structural perspective, the MFS transporter fold is characterized by the interactions between the two global N- and C-terminal bundles and contains several three-helix inverted repeat units that are thought to play a critical role during solute transport across the membrane (Radestock and Forrest, 2011). These individual inverted three-transmembrane helix elements are repeated four times in MFS transporters with 12-TMSs. It remains unclear to what extent the inverted three-helix repeat structural motif is conserved in MFS antimicrobial efflux pumps with 14-TMSs (Brawley et al., 2022; Kumar et al., 2021).

One major hallmark of the MFS is the shared conservation of amino acid sequence motifs (Kakarla et al., 2017b). The shared sequences’ similarities predicted that transporters of the MFS shared similar structures and, consequently, the superfamily members shared a common evolutionary origin (Mitchell, 1991). The functional importance of shared signature sequences has been established in antimicrobial transporters of the MFS (Varela et al., 1995; Yamaguchi et al., 1992). Motif A resides in the cytoplasmic loop between helices two and three and is thought to stabilize the outward-facing conformation (Jiang et al., 2013). Motif C, known as the antiporter motif (Varela et al., 1995), resides in the fifth TMS of antimicrobial efflux pumps and plays a critical role as a conformational switch (Nagarathinam et al., 2018).

Another common feature that MFS transporters share is a large central cavity formed by residues tied to the two bundles, which are known to functionally interact through a so-called alternating substrate-ion access process on either side of the membrane during transport (Henderson, 1991). In addition to its role as a substrate accessing path to the binding site, the central cavity has been proposed to form the conduit by which the energy of the ion-motive force, such as that generated by proton gradients across the membrane during respiration, is used to drive the transport of substrates against the concentration gradient, as predicted by Mitchell and his proton sink (Mitchell, 1991).

The antimicrobial antiport transport cycle in bacteria is driven by the ion-motive force, an energy mode associated with changes in transporter conformations and gating during efflux across the membrane (Drew et al., 2021). Over decades, physiological data combined with structure-function analyses often involving mutagenesis and determination of crystal structures at various stages of the transport cycle have culminated in an overall consensus of the events that occur during ion-driven efflux of antimicrobial substrates (Drew et al., 2021; Henderson, 1991; Krämer, 1994; Poolman and Konings, 1993; Radestock and Forrest, 2011), Fig. 3. A cell undergoing respiration generates the electrochemical potential energy (ion-motive force) called chemiosmosis, which provides active efflux energy (Mitchell, 1991). In stage one, the antimicrobial efflux pump is empty, facing outward, and open to externally located ions (Yin et al., 2006). In stage two, the energizing ion binds the outward-facing open transporter, generating an occluded carrier state without a drug substrate (Jiang et al., 2013; Kumar et al., 2021; Nagarathinam et al., 2018; Schaedler and Van Veen, 2010). Stage 3 involves a conformational change of the efflux pump to produce a cytoplasmic-facing (inward) open conformation state that binds with the antimicrobial substrate (Xiao et al., 2021). Stage 4 involves binding the drug substrate and releasing the ion (Heng et al., 2015; Schaedler and Van Veen, 2010; Xiao et al., 2021). The binding order of ion and substrate has not been fully determined and remains a point of contention. It is widely appreciated that substrate binding generates stage 5, an occupied occluded conformational state (Krämer, 1994; Poolman and Konings, 1993; Zomot et al., 2018). In stage 6, a conformational alteration results in the exposure of the bound substrate to an outward-facing occluded conformation to decrease substrate-binding affinity, thereby releasing the antimicrobial to the periplasmic or extracellular milieu (Debruycker et al., 2020; Kumar et al., 2021). The release of the antimicrobial returns the transporter to its original outward-facing open stage, ready to repeat the transport cycle (Krämer, 1994; Poolman and Konings, 1993).

**Fig. 3:**
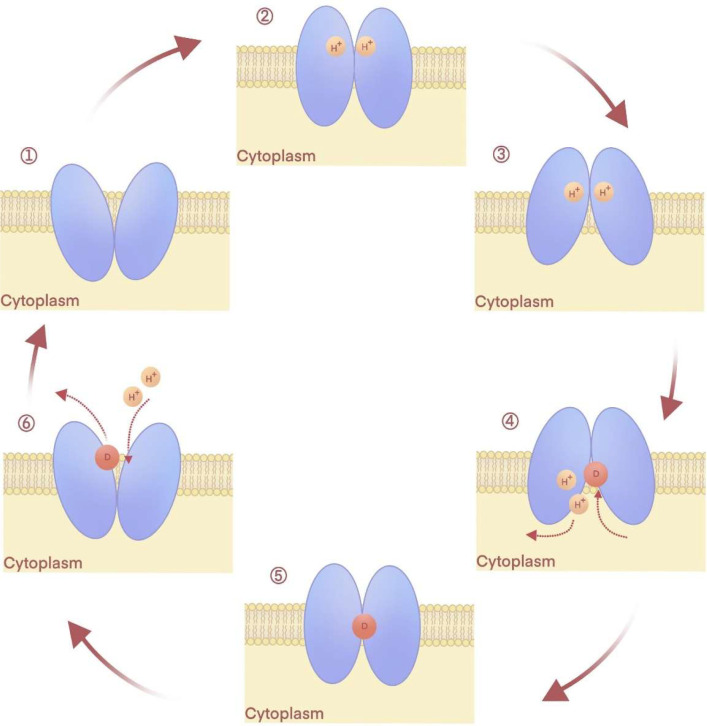
MFS drug substrate/ion antiport mechanism across the membrane. Stage 1 shows an empty externally-facing (open outward) transporter. Stage 2 is characterized by a bound ion and is considered occluded and empty of the substrate. Stage 3 is an inward-facing open conformation. Stage 4 is an inward-facing occluded state after ion release and binding of the drug. Stage 5 is a substrate-bound occluded conformation. Stage 6 is an occluded outward-facing conformation that releases the substrate (Dang et al., 2010; Xiao et al., 2021).

The LmrP multidrug transporter protein structure from L. *lactis* showed that embedded anionic lipids stabilized the substrate-bound conformation in an outward-facing open state (Debruycker et al., 2020). A combination of molecular simulation dynamics and site-directed mutagenesis was used to suggest that lipids in the LmrP substrate binding pocket made it malleable, providing a hydrophobic microenvironment conducive to conferring multiple substrate specificities.

In an interesting report on MdfA, investigators used molecular simulation dynamics during outward and inward-facing conformational changes to identify novel salt bridges (Ying Li and Ge, 2023). They found that the antimicrobial efflux and resistance levels were enhanced when specific new salt bridges were engineered. In an inward-facing state, a salt bridge was formed between Asp-52 of motif A and Lys-369. In contrast, the outward-facing conformation harbored a salt bridge between Glu-136 and Arg-336. However, when Glu-136 was altered to Asp and Lys-346 was changed to Arg, the artificial salt bridge showed stabilization, enhancing antimicrobial transport and resistance.

Another study used the MdfA structure for homology modeling of the putative MFS transporter Rv1634 from *Mycobacterium tuberculosis* to predict a drug binding site and the substrate translocation channel through the central cavity, suggesting a possible pathway of a drug through the pump across the membrane (Singh and Akhter, 2022). Thus, the methodological approach of structure prediction using homology modeling, prediction of drug binding pockets, and tunnel pathway detection represents a useful strategy for analyses of new and extant MFS transporter physiology. Predictive studies like this can be readily tested by drug and ion transport studies across the membrane and yield specific molecular targets for modulation.

One recent study showed that in the MFS spinster lipid exporter, *Hn*Spns, from the bacterium *Hyphomonas neptunium*, two conserved amino acid residues that become protonated at various conformational stages during efflux were identified (Dastvan et al., 2022). A conserved salt bridge forms between Asp-60 of the motif A signature sequence on TMS-2 and Arg-289 on TMS-7 to form a stabilized outward-facing closed conformation in *Hn*Spns.

### 4.4 Recent modulation studies

Multidrug efflux pumps of the MFS represent good targets for modulation to restore the efficacy of antimicrobial agents that are compromised by bacterial resistance (Kumar et al., 2016a). However, many studies on efflux pump modulation involve non-specific effects that are toxic to individuals or require non-physiological concentrations to mediate transport inhibition. One promising strategy consists of using natural modulators targeting drug efflux pumps instead of respiratory chain machinery, which can cause toxicity (Shrestha et al., 2018). Another promising approach can focus on particular pathogenic species, such as *S. aureus* (Lekshmi et al., 2018; Floyd et al., 2010) and *V. cholerae* (Stephen et al., 2022). Previously, our laboratory reported reducing antimicrobial resistance by inhibiting the MFS multidrug efflux pump LmrS from a methicillin-resistant *S. aureus* by the bioactive agent in cumin spice from *Cuminum cyminum* (Kakarla et al., 2017a; Smith et al., 2009). More recently, LmrS was demonstrated to be modulated by calcium ions (Nava et al., 2020) and the peptide antibiotic plantaricin A (Meng et al., 2022). Further, the expression of LmrS can be regulated by the TetR21 repressor protein (Truong-Bolduc et al., 2017). Thus, studies of the LmrS multidrug efflux pump can serve as a useful model system for virulence and modulation to reduce clinical conditions that foster infection.

Similarly, our laboratory demonstrated that the bioactive agent allyl sulfide from *Allium sativum* not only inhibited drug transport of the MFS multidrug efflux pump EmrD from *V. cholerae* but that garlic extract worked synergistically to enhance the efficacy of traditional antimicrobial agents in host bacteria (Smith et al., 2009; Bruns et al., 2017). This latter study represents the useful strategy of drug combination therapy toward reestablishing the effectiveness of clinical antimicrobials previously compromised by pathogens harboring MFS multidrug efflux pump systems.

A recent systematic study was reported of the effects of an antibody fragment on the NorA multidrug efflux pump structure from *S. aureus* (Brawley et al., 2022). The transporter was bound to antigen-binding fragment (Fab) domains, inhibiting the drug efflux activity. The new finding is important in establishing Fab peptides as a suitable strategy for inhibiting multidrug resistance mediated by antimicrobial transporters of the MFS from bacterial pathogens.

More recently, using molecular dynamics simulations, the transport of ciprofloxacin was inhibited by the naturally-occurring compound berberine by its binding to both inward- and outward-facing conformations of the MdfA multidrug efflux pump of *E. coli* (Y. Li and Ge, 2023). Thus, the new study demonstrates that elements of the transport machinery responsible for mediating conformational changes during transport constitute suitable targets for effective modulation (Y. Li and Ge, 2023). Once a clear understanding of the transport processes conducted by MFS antimicrobial transporters is attained, it can be possible to determine a unifying paradigm for addressing infection by serious bacterial pathogens.

## 5 Directions for future investigation

The structural nature of the MFS multidrug efflux pump systems from Gram-positive bacteria remains unclear and needs attention. Until recently, the nature of the MFS fold structural arrangement had been obscure in MFS proteins predicted to harbor 14-TMSs (Forrest et al., 2008). It is poorly understood to what extent the protein structures of well-studied antimicrobial transporters like QacA from *S. aureus* pathogens are related to the known structures of MdfA, YajR, LmrP, NorA, and EmrD [62]. Along these lines, the extent to which the protein structures and mechanisms of transport across the membrane of the MFS antimicrobial efflux pumps are shared in bacterial pathogens needs to be better understood.

A key strategy for addressing multidrug resistance modulation involves identifying the structural and conformational elements dictated by highly conserved sequence motifs that influence the rocker-switch system of antimicrobial efflux (Law et al., 2008; Kakarla et al., 2017b; Varela and Kumar, 2019). It is anticipated that efforts to understand better the functional roles that individual conserved residues play in shared signature sequence motifs can provide novel insights into the processes involved in the overall antimicrobial transport cycle.

While new insights into the molecular basis for the efflux of multiple structurally different antimicrobial agents have been reported, we still need to understand what mechanisms or physiological systems play roles in determining poly-substrate specificities assigned to individual MFS pumps. For each antimicrobial pump of the MFS, the molecular natures that determine the substrate selection profiles require our attention. As such, we still need to fully understand how multidrug specificity is dictated for each transporter while, at the same time, leakage of water or ions is prevented.

One neglected avenue is the study of the relationships between individual efflux pump inhibitors and putative enhancement of antimicrobial activities when present in combinations with clinical antimicrobial agents to mediate synergy during clinical treatment. Recently, it was shown that a combination of norfloxacin with the 1,8-naphthyridine sulfonamides demonstrated synergy against *S. aureus*, providing a potentially non-toxic approach toward treating infection (Oliveira-Tintino et al., 2021). Studies like this represent a good paradigm for future work. While many efflux pump inhibitors for bacterial multidrug efflux pumps of the MFS have been discovered (Varela et al., 2023), few of these modulators have been translated into effective therapeutics for infection caused by bacterial pathogens. The reasons for this apparent disparity remain unclear. Future studies are necessary to translate these studies into effective therapy for infection caused by serious bacterial pathogens.

## Funding

The studies reported from our laboratories and considered in this publication were supported in part by Faculty Research and Instructional Development grants awarded by ENMU and research grants awarded by the National Institute of the General Medical Sciences (P20GM103451) from the National Institutes of Health, plus the U.S. Department of Education, HSI-STEM program (P031C110114).

## CRediT authorship contribution statement

**Manjusha Lekshmi:** Writing – original draft, Writing – review & editing. **Anely Ortiz-Alegria:** Writing – original draft, Writing – review & editing. **Sanath Kumar:** Conceptualization, Writing – original draft, Writing – review & editing. **Manuel F. Varela:** Conceptualization, Writing – original draft, Writing – review & editing.

## Declaration of competing interest

The authors declare that they have no known competing financial interests or personal relationships that could have appeared to influence the work reported in this paper

## References

[bib0001] Aldred K.J., Kerns R.J., Osheroff N. (2014). Mechanism of quinolone action and resistance. Biochemistry.

[bib0002] Baker J., Wright S.H., Tama F. (2012). Simulations of substrate transport in the multidrug transporter EmrD. Proteins..

[bib0003] Blair J.M.A., Webber M.A., Baylay A.J., Ogbolu D.O., Piddock L.J.V. (2015). Molecular mechanisms of antibiotic resistance. Nat. Rev. Microbiol..

[bib0004] Brawley D.N., Sauer D.B., Li J., Zheng X., Koide A., Jedhe G.S., Suwatthee T., Song J., Liu Z., Arora P.S., Koide S., Torres V.J., Wang D.N., Traaseth N.J. (2022). Structural basis for inhibition of the drug efflux pump NorA from *Staphylococcus aureus*. Nat. Chem. Biol..

[bib0005] Brown M.H., Paulsen I.T., Skurray R.A. (1999). The multidrug efflux protein NorM is a prototype of a new family of transporters. Mol. Microbiol..

[bib0006] Bruns M.M., Kakarla P., Floyd J.T., Mukherjee M.M., Ponce R.C., Garcia J.A., Ranaweera I., Sanford L.M., Hernandez A.J., Willmon T.M. (2017). Modulation of the multidrug efflux pump EmrD-3 from *Vibrio cholerae* by *Allium sativum* extract and the bioactive agent allyl sulfide plus synergistic enhancement of antimicrobial susceptibility by *A. sativum* extract. Arch. Microbiol..

[bib0007] Bush K. (2018). Past and Present Perspectives on β-Lactamases. Antimicrob. Agents Chemother..

[bib0008] Cantón, R., 2008. Epidemiology and evolution of beta-lactamases. Evolutionary biology of bacterial and fungal pathogens 249–270. 10.1128/9781555815639.ch22.

[bib0009] Costa S.S., Viveiros M., Amaral L., Couto I. (2013). Multidrug Efflux Pumps in *Staphylococcus aureus*: an Update. Open. Microbiol. J..

[bib0010] Dang S., Sun L., Huang Y., Lu F., Liu Y., Gong H., Wang J., Yan N. (2010). Structure of a fucose transporter in an outward-open conformation. Nature.

[bib0011] Dashtbani-Roozbehani A., Brown M.H. (2021). Efflux pump mediated antimicrobial resistance by staphylococci in health-related environments: challenges and the quest for inhibition. Antibiotics. (Basel).

[bib0012] Dastvan R., Rasouli A., Dehghani-Ghahnaviyeh S., Gies S., Tajkhorshid E. (2022). Proton-driven alternating access in a spinster lipid transporter. Nat. Commun..

[bib0013] Davidson A.L., Dassa E., Orelle C., Chen J. (2008). Structure, function, and evolution of bacterial ATP-binding cassette systems. Microbiol. Mol. Biol. Rev.

[bib0014] Debruycker V., Hutchin A., Masureel M., Ficici E., Martens C., Legrand P., Stein R.A., McHaourab H.S., Faraldo-Gomez J.D., Remaut H., Govaerts C. (2020). An embedded lipid in the multidrug transporter LmrP suggests a mechanism for polyspecificity. Nat. Struct. Mol. Biol..

[bib0015] Drew D., North R.A., Nagarathinam K., Tanabe M. (2021). Structures and general transport mechanisms by the major facilitator superfamily (MFS). Chem. Rev..

[bib0016] Floyd J.L., Smith K.P., Kumar S.H., Floyd J.T., Varela M.F. (2010). LmrS is a multidrug efflux pump of the major facilitator superfamily from *Staphylococcus aureus*. Antimicrob. Agents Chemother.

[bib0017] Forrest L.R., Zhang Y.-W., Jacobs M.T., Gesmonde J., Xie L., Honig B.H., Rudnick G. (2008). Mechanism for alternating access in neurotransmitter transporters. Proc. Natl. Acad. Sci. u S. a.

[bib0018] Fratamico P.M., DebRoy C., Liu Y., Needleman D.S., Baranzoni G.M., Feng P. (2016). Advances in molecular serotyping and subtyping of *Escherichia coli*. Front. Microbiol..

[bib0019] Garneau-Tsodikova S., Labby K.J. (2016). Mechanisms of resistance to aminoglycoside antibiotics: overview and perspectives. Medchemcomm..

[bib0020] Gaurav A., Bakht P., Saini M., Pandey S., Pathania R. (2023). Role of bacterial efflux pumps in antibiotic resistance, virulence, and strategies to discover novel efflux pump inhibitors. Microbiology (Reading).

[bib0021] Georgopapadakou N.H. (1993). Penicillin-binding proteins and bacterial resistance to β-lactams. Antimicrob. Agents Chemother.

[bib0022] Gomes T.A.T., Elias W.P., Scaletsky I.C.A., Guth B.E.C., Rodrigues J.F., Piazza R.M.F., Ferreira L.C.S., Martinez M.B. (2016). Diarrheagenic *Escherichia coli*. Braz. J. Microbiol. 47 Suppl.

[bib0023] Griffith J.K., Baker M.E., Rouch D.A., Page M.G., Skurray R.A., Paulsen I.T., Chater K.F., Baldwin S.A., Henderson P.J. (1992). Membrane transport proteins: implications of sequence comparisons. Curr. Opin. Cell Biol..

[bib0024] Hall B.G., Barlow M. (2005). Revised Ambler classification of β-lactamases. J. Antimicrob. Chemother.

[bib0025] Hassan K.A., Liu Q., Elbourne L.D.H., Ahmad I., Sharples D., Naidu V., Chan C.L., Li L., Harborne S.P.D., Pokhrel A., Postis V.L.G., Goldman A., Henderson P.J.F., Paulsen I.T. (2018). Pacing across the membrane: the novel PACE family of efflux pumps is widespread in Gram-negative pathogens. Res. Microbiol..

[bib0026] Hassan K.A., Liu Q., Henderson P.J.F., Paulsen I.T. (2015). Homologs of the *Acinetobacter baumannii* AceI transporter represent a new family of bacterial multidrug efflux systems. mBio.

[bib0027] Hassan K.A., Naidu V., Edgerton J.R., Mettrick K.A., Liu Q., Fahmy L., Li L., Jackson S.M., Ahmad I., Sharples D., Henderson P.J.F., Paulsen I.T. (2019). Short-chain diamines are the physiological substrates of PACE family efflux pumps. Proc. Natl. Acad. Sci. u S. a.

[bib0028] He G.-X., Kuroda T., Mima T., Morita Y., Mizushima T., Tsuchiya T. (2004). An H^+^-coupled multidrug efflux pump, PmpM, a member of the MATE family of transporters, from Pseudomonas aeruginosa. J. Bacteriol..

[bib0029] Henderson P.J. (1991). Studies of translocation catalysis. Biosci. Rep..

[bib0030] Heng J., Zhao Y., Liu M., Liu Y., Fan J., Wang X., Zhang X.C. (2015). Substrate-bound structure of the *E. coli* multidrug resistance transporter MdfA. Cell Res..

[bib0031] Huda M.N., Morita Y., Kuroda T., Mizushima T., Tsuchiya T. (2001). Na^+^-driven multidrug efflux pump VcmA from *Vibrio cholerae* non-O1, a non-halophilic bacterium. FEMS Microbiol. Lett..

[bib0032] Hvorup R.N., Winnen B., Chang A.B., Jiang Y., Zhou X.F., Saier M.H. (2003). The multidrug/oligosaccharidyl-lipid/polysaccharide (MOP) exporter superfamily. Eur. J. Biochem..

[bib0033] Jiang D., Zhao Y., Fan J., Liu X., Wu Y., Feng W., Zhang X.C. (2014). Atomic resolution structure of the *E. coli* YajR transporter YAM domain. Biochem. Biophys. Res. Commun..

[bib0034] Jiang D., Zhao Y., Wang X., Fan J., Heng J., Liu X., Feng W., Kang X., Huang B., Liu J., Zhang X.C. (2013). Structure of the YajR transporter suggests a transport mechanism based on the conserved motif A. Proc. Natl. Acad. Sci. U.S.A..

[bib0035] Jubeh B., Breijyeh Z., Karaman R. (2020). Resistance of gram-positive bacteria to current antibacterial agents and overcoming approaches. Molecules..

[bib0036] Kakarla P., Floyd J., Mukherjee M., Devireddy A.R., Inupakutika M.A., Ranweera I., Kc R., Shrestha U., Cheeti U.R., Willmon T.M. (2017). Inhibition of the multidrug efflux pump LmrS from *Staphylococcus aureus* by cumin spice *Cuminum cyminum*. Arch. Microbiol..

[bib0037] Kakarla P., KC R., Shrestha U., Ranaweera I., Mukherjee M.M., Willmon T.M., Hernandez A.J., Barr S.R., Varela M.F. (2017). Drug Resistance in Bacteria, Fungi, Malaria, and Cancer.

[bib0039] Kaper J.B., Nataro J.P., Mobley H.L.T. (2004). Pathogenic *Escherichia coli*. Nat. Rev. Microbiol..

[bib0040] Kapoor G., Saigal S., Elongavan A. (2017). Action and resistance mechanisms of antibiotics: a guide for clinicians. J. Anaesthesiol. Clin. Pharmacol..

[bib0041] Kaye K.S., Pogue J.M. (2015). Infections caused by resistant gram-negative bacteria: epidemiology and management. Pharmacotherapy..

[bib0042] Kim J., Cater R.J., Choy B.C., Mancia F. (2021). Structural insights into transporter-mediated drug resistance in infectious diseases. J. Mol. Biol..

[bib0043] Koulenti D., Xu E., Yin Sum Mok I., Song A., Karageorgopoulos D.E., Armaganidis A., Lipman J., Tsiodras S. (2019). Novel Antibiotics for Multidrug-Resistant Gram-Positive Microorganisms. Microorganisms..

[bib0044] Krämer R. (1994). Functional principles of solute transport systems: concepts and perspectives. Biochim. Biophys. Acta.

[bib0045] Kumar S., Athreya A., Gulati A., Nair R.M., Mahendran I., Ranjan R., Penmatsa A. (2021). Structural basis of inhibition of a transporter from *Staphylococcus aureus*, NorC, through a single-domain camelid antibody. Commun. Biol..

[bib0046] Kumar S., He G., Kakarla P., Shrestha U., KC R., Ranaweera I., Mark Willmon T., Barr S., Hernandez A., Varela M. (2016). Bacterial multidrug efflux pumps of the major facilitator superfamily as targets for modulation. Infect. Disord.-Drug Targets (Formerly Curr. Drug Targets-Infect. Disord.).

[bib0047] Kumar S., Lekshmi M., Parvathi A., Ojha M., Wenzel N., Varela M.F. (2020). Functional and Structural Roles of the Major Facilitator Superfamily Bacterial Multidrug Efflux Pumps. Microorganisms..

[bib0048] Kumar S., Mahendran I., Athreya A., Ranjan R., Penmatsa A. (2020). Isolation and structural characterization of a Zn^2+^-bound single-domain antibody against NorC, a putative multidrug efflux transporter in bacteria. J. Biol. Chem..

[bib0049] Kumar S., Ranjana K., Sanford L.M., Hernandez A.J., Kakarla P., Varela M.F. (2016). Structural and functional roles of two evolutionarily conserved amino acid sequence motifs within solute transporters of the major facilitator superfamily. Trends. Cell Mol. Biol..

[bib0050] Kumar S., Varela M.F., Méndez-Vilas A. (2013). Microbial Pathogens and Strategies for Combating Them: Science, Technology and Education.

[bib0051] Kuroda T., Tsuchiya T. (2009). Multidrug efflux transporters in the MATE family. Biochim. Biophys. Acta.

[bib0052] Law C.J., Maloney P.C., Wang D.N. (2008). Ins and outs of major facilitator superfamily antiporters. Annu. Rev. Microbiol..

[bib0053] Leclercq R. (2002). Mechanisms of resistance to macrolides and lincosamides: nature of the resistance elements and their clinical implications. Clin. Infect. Dis..

[bib0054] Lekshmi M., Ammini P., Adjei J., Sanford L.M., Shrestha U., Kumar S., Varela M.F. (2018). Modulation of antimicrobial efflux pumps of the major facilitator superfamily in *Staphylococcus aureus*. AIMS. Microbiol..

[bib0056] Levy S.B. (2002). Active efflux, a common mechanism for biocide and antibiotic resistance. J. Appl. Microbiol..

[bib0057] Li Ying, Ge X. (2023). Enhanced internal ionic interaction of MFS efflux pump MdfA contributes to its elevated antibiotic export. Phys. Chem. Chem. Phys..

[bib0058] Li Y., Ge X. (2023). Role of berberine as a potential efflux pump inhibitor against MdfA from *Escherichia coli: in vitro* and *in silico* studies. Microbiol. Spectr..

[bib0059] Liu M., Heng J., Gao Y., Wang X. (2016). Crystal structures of MdfA complexed with acetylcholine and inhibitor reserpine. Biophys. Rep..

[bib0060] Maiden M.C., Davis E.O., Baldwin S.A., Moore D.C., Henderson P.J. (1987). Mammalian and bacterial sugar transport proteins are homologous. Nature.

[bib0061] Marger M.D., Saier M.H. (1993). A major superfamily of transmembrane facilitators that catalyse uniport, symport and antiport. Trends Biochem. Sci..

[bib0062] Martirosov D.M., Lodise T.P. (2016). Emerging trends in epidemiology and management of infections caused by carbapenem-resistant Enterobacteriaceae. Diagn. Microbiol. Infect. Dis..

[bib0063] Meng F., Nie T., Lyu Y., Lyu F., Bie X., Lu Y., Zhao M., Lu Z. (2022). Plantaricin A reverses resistance to ciprofloxacin of multidrug-resistant *Staphylococcus aureus* by inhibiting efflux pumps. Environ. Microbiol..

[bib0064] Mitchell P. (1991). Foundations of vectorial metabolism and osmochemistry. Biosci. Rep..

[bib0065] Morita Y., Kataoka A., Shiota S., Mizushima T., Tsuchiya T. (2000). NorM of *vibrio parahaemolyticus* is an Na^+^-driven multidrug efflux pump. J. Bacteriol..

[bib0066] Morita Y., Kodama K., Shiota S., Mine T., Kataoka A., Mizushima T., Tsuchiya T. (1998). NorM, a putative multidrug efflux protein, of *Vibrio parahaemolyticus* and its homolog in *Escherichia coli*. Antimicrob. Agents Chemother.

[bib0067] Murray C.J.L., Ikuta K.S., Sharara F. (2022). Global burden of bacterial antimicrobial resistance in 2019: a systematic analysis. Lancet.

[bib0068] Nagarathinam K., Nakada-Nakura Y., Parthier C., Terada T., Juge N., Jaenecke F., Liu K., Hotta Y., Miyaji T., Omote H., Iwata S., Nomura N., Stubbs M.T., Tanabe M. (2018). Outward open conformation of a major facilitator superfamily multidrug/H^+^ antiporter provides insights into switching mechanism. Nat. Commun..

[bib0069] Nava A.R., Mauricio N., Sanca A.J., Domínguez D.C. (2020). Evidence of calcium signaling and modulation of the LmrS multidrug resistant efflux pump activity by Ca^2+^ Ions in *S. aureus*. Front. Microbiol..

[bib0070] Neyfakh A.A. (1992). The multidrug efflux transporter of *Bacillus subtilis* is a structural and functional homolog of the *Staphylococcus* NorA protein. Antimicrob. Agents Chemother.

[bib0071] Nguyen T.H.T., Nguyen H.D., Le M.H., Nguyen T.T.H., Nguyen T.D., Nguyen D.L., Nguyen Q.H., Nguyen T.K.O., Michalet S., Dijoux-Franca M.-G., Pham H.N. (2023). Efflux pump inhibitors in controlling antibiotic resistance: outlook under a heavy metal contamination context. Molecules..

[bib0072] Nikaido H. (2011). Structure and mechanism of RND-type multidrug efflux pumps. Adv. Enzymol. Relat. Areas Mol. Biol..

[bib0073] Nikaido H. (2003). Molecular basis of bacterial outer membrane permeability revisited. Microbiol. Mol. Biol. Rev. : MMBR.

[bib0074] Nikaido H., Takatsuka Y. (2009). Mechanisms of RND multidrug efflux pumps. Biochim. Biophys. Acta.

[bib0075] Oliveira-Tintino C.D.de M., Muniz D.F., Barbosa C.R.dos S., Pereira R.L.S., Begnini I.M., Rebelo R.A., Silva L.E.da, Mireski S.L., Nasato M.C., Krautler M.I.L., Pereira P.S., Costa J.G.M.da, Rodrigues F.F.G., Teixeira A.M.R., Ribeiro-Filho J., Tintino S.R., de Menezes I.R.A., Coutinho H.D.M., Silva T.G.da (2021). The 1,8-naphthyridines sulfonamides are NorA efflux pump inhibitors. J. Glob. Antimicrob. Resist..

[bib0076] O’Neill J. (2014). Antimicrobial resistance: tackling a crisis for the health and wealth of nations. The review on antimicrobial resistance.

[bib0077] Pages J.M., James C.E., Winterhalter M. (2008). The porin and the permeating antibiotic: a selective diffusion barrier in gram-negative bacteria. Nat. Rev. Microbiol..

[bib0078] Pao S.S., Paulsen I.T., Saier M.H. (1998). Major facilitator superfamily. Microbiol. Mol. Biol. Rev..

[bib0079] Pasqua M., Grossi M., Scinicariello S., Aussel L., Barras F., Colonna B., Prosseda G. (2019). The MFS efflux pump EmrKY contributes to the survival of *Shigella* within macrophages. Sci. Rep..

[bib0080] Paterson D.L., Bonomo R.A. (2005). Extended-spectrum β-lactamases: a clinical update. Clin. Microbiol. Rev..

[bib0081] Poolman B., Konings W.N. (1993). Secondary solute transport in bacteria. Biochim. Biophys. Acta.

[bib0082] Prajapati J.D., Kleinekathöfer U., Winterhalter M. (2021). How to enter a bacterium: bacterial porins and the permeation of antibiotics. Chem. Rev..

[bib0083] Queenan A.M., Bush K. (2007). Carbapenemases: the Versatile β-Lactamases. Clin. Microbiol. Rev..

[bib0084] Radestock S., Forrest L.R. (2011). The alternating-access mechanism of MFS transporters arises from inverted-topology repeats. J. Mol. Biol..

[bib0085] Ranaweera I., Shrestha U., Ranjana K.C., Kakarla P., Willmon T.M., Hernandez A.J., Mukherjee M.M., Barr S.R., Varela M.F. (2015). Structural comparison of bacterial multidrug efflux pumps of the major facilitator superfamily. Trends. Cell Mol. Biol..

[bib0086] Russo T.A., Johnson J.R. (2000). Proposal for a new inclusive designation for extraintestinal pathogenic isolates of *Escherichia coli*: exPEC. J. Infect. Dis..

[bib0087] Saier M.H., Beatty J.T., Goffeau A., Harley K.T., Heijne W.H., Huang S.C., Jack D.L., Jähn P.S., Lew K., Liu J., Pao S.S., Paulsen I.T., Tseng T.T., Virk P.S. (1999). The major facilitator superfamily. J. Mol. Microbiol. Biotechnol..

[bib0088] Saier M.H. (1998). Molecular phylogeny as a basis for the classification of transport proteins from bacteria, archaea and eukarya. Adv. Microb. Physiol..

[bib0089] Saier M.H., Paulsen I.T., Sliwinski M.K., Pao S.S., Skurray R.A., Nikaido H. (1998). Evolutionary origins of multidrug and drug-specific efflux pumps in bacteria. FASEB J..

[bib0090] Saier M.H., Reddy V.S., Moreno-Hagelsieb G., Hendargo K.J., Zhang Y., Iddamsetty V., Lam K.J.K., Tian N., Russum S., Wang J., Medrano-Soto A. (2021). The transporter classification database (TCDB): 2021 update. Nucleic Acids Res..

[bib0091] Sawa T., Kooguchi K., Moriyama K. (2020). Molecular diversity of extended-spectrum β-lactamases and carbapenemases, and antimicrobial resistance. J. Intensive Care.

[bib0092] Schaedler T.A., Van Veen H.W. (2010). A flexible cation binding site in the multidrug major facilitator superfamily transporter LmrP is associated with variable proton coupling. FASEB J..

[bib0093] Schwarz S., Kehrenberg C., Doublet B., Cloeckaert A. (2004). Molecular basis of bacterial resistance to chloramphenicol and florfenicol. FEMS Microbiol. Rev..

[bib0094] Shi Y. (2013). Common folds and transport mechanisms of secondary active transporters. Annu Rev. Biophys..

[bib0095] Shoaib M., Aqib A.I., Muzammil I., Majeed N., Bhutta Z.A., Kulyar M.F.-A., Fatima M., Zaheer C.-N.F., Muneer A., Murtaza M., Kashif M., Shafqat F., Pu W. (2023). MRSA compendium of epidemiology, transmission, pathophysiology, treatment, and prevention within one health framework. Front. Microbiol..

[bib0096] Shrestha U., Lekshmi M., Kumar S., Adjei J., Jones K.M., Hernandez A.J., Sanford L.M., Varela M.F. (2018). Bioactive agents as modulators of multidrug efflux pumps of the major facilitator super family in key bacterial pathogens. Curr. Trends Microbiol..

[bib0097] Sievert D.M., Ricks P., Edwards J.R., Schneider A., Patel J., Srinivasan A., Kallen A., Limbago B., Fridkin S. (2013). Antimicrobial-resistant pathogens associated with healthcare-associated infections: summary of data reported to the National Healthcare Safety Network at the Centers for Disease Control and Prevention, 2009-2010. Infect. Control Hosp. Epidemiol..

[bib0098] Sigal N., Lewinson O., Wolf S.G., Bibi E. (2007). *E. coli* multidrug transporter MdfA is a monomer. Biochemistry.

[bib0099] Singh G., Akhter Y. (2022). Molecular insights into the differential efflux mechanism of Rv1634 protein, a multidrug transporter of major facilitator superfamily in *Mycobacterium tuberculosis*. Proteins..

[bib0100] Sirot J., Chanal C., Petit A., Sirot D., Labia R., Gerbaud G. (1988). *Klebsiella pneumoniae* and other Enterobacteriaceae producing novel plasmid-mediated β-lactamases markedly active against third-generation cephalosporins: epidemiologic studies. Rev. Infect. Dis..

[bib0101] Smith K.P., Kumar S., Varela M.F. (2009). Identification, cloning, and functional characterization of EmrD-3, a putative multidrug efflux pump of the major facilitator superfamily from *Vibrio cholerae* O395. Arch. Microbiol..

[bib0102] Stephen J., Lekshmi M., Ammini P., Kumar S.H., Varela M.F. (2022). Membrane efflux pumps of pathogenic *vibrio* species: role in antimicrobial resistance and virulence. Microorganisms..

[bib0103] Stephen J., Salam F., Lekshmi M., Kumar S.H., Varela M.F. (2023). The major facilitator superfamily and antimicrobial resistance efflux pumps of the ESKAPEE pathogen *Staphylococcus aureus*. Antibiotics.

[bib0104] Sugawara E., Nikaido H. (2012). OmpA Is the principal nonspecific slow porin of *Acinetobacter baumannii*. J. Bacteriol..

[bib0105] Truong-Bolduc Q.C., Wang Y., Chen C., Hooper D.C. (2017). Transcriptional regulator TetR21 controls the expression of the *Staphylococcus aureus* LmrS efflux pump. Antimicrob. Agents Chemother.

[bib0106] Turner N.A., Sharma-Kuinkel B.K., Maskarinec S.A., Eichenberger E.M., Shah P.P., Carugati M., Holland T.L., Fowler V.G. (2019). Methicillin-resistant *Staphylococcus aureus*: an overview of basic and clinical research. Nat. Rev. Microbiol..

[bib0107] Varela M.F. (2019). Antibiotic Drug Resistance.

[bib0108] Varela M.F., Kumar S. (2019). Strategies for discovery of new molecular targets for anti-infective drugs. Curr. Opin. Pharmacol..

[bib0109] Varela M.F., Sansom C.E., Griffith J.K. (1995). Mutational analysis and molecular modelling of an amino acid sequence motif conserved in antiporters but not symporters in a transporter superfamily. Mol. Membr. Biol..

[bib0110] Varela M.F., Stephen J., Bharti D., Lekshmi M., Kumar S. (2023). Inhibition of multidrug efflux pumps belonging to the major facilitator superfamily in bacterial pathogens. Biomedicines..

[bib0111] Varela M.F., Stephen J., Lekshmi M., Ojha M., Wenzel N., Sanford L.M., Hernandez A.J., Parvathi A., Kumar S.H. (2021). Bacterial resistance to antimicrobial agents. Antibiotics.

[bib0112] Venter H., Mowla R., Ohene-Agyei T., Ma S. (2015). RND-type drug efflux pumps from Gram-negative bacteria: molecular mechanism and inhibition. Front. Microbiol..

[bib0113] West I.C. (1980). Energy coupling in secondary active transport. Biochim. Biophys. Acta.

[bib0114] WHO (World Health Organization), 2023. Antimicrobial resistance [WWW Document]. URL https://www.who.int/news-room/fact-sheets/detail/antimicrobial-resistance (accessed 6.18.23).

[bib0115] Wright G.D. (2005). Bacterial resistance to antibiotics: enzymatic degradation and modification. Adv. Drug Deliv. Rev..

[bib0116] Wright Gerard D. (2005). Bacterial resistance to antibiotics: enzymatic degradation and modification. Adv. Drug Deliv. Rev..

[bib0117] Xiao Q., Sun B., Zhou Y., Wang C., Guo L., He J., Deng D. (2021). Visualizing the nonlinear changes of a drug-proton antiporter from inward-open to occluded state. Biochem. Biophys. Res. Commun..

[bib0118] Yamaguchi A., Someya Y., Sawai T. (1992). Metal-tetracycline/H^+^ antiporter of *Escherichia coli* encoded by transposon Tn10. The role of a conserved sequence motif, GXXXXRXGRR, in a putative cytoplasmic loop between helices 2 and 3. J. Biol. Chem..

[bib0119] Yang X., Goswami S., Gorityala B.K., Domalaon R., Lyu Y., Kumar A., Zhanel G.G., Schweizer F. (2017). A tobramycin vector enhances synergy and efficacy of efflux pump inhibitors against multidrug-resistant gram-negative bacteria. J. Med. Chem..

[bib0120] Yin Y., He X., Szewczyk P., Nguyen T., Chang G. (2006). Structure of the multidrug transporter EmrD from *Escherichia coli*. Science.

[bib0121] Yun C.S., Moon B.-Y., Hwang M.-H., Lee S.-K., Ku B.-K., Lee K. (2023). Characterization of the pathogenicity of extraintestinal pathogenic *Escherichia coli* isolates from pneumonia-infected lung samples of dogs and cats in South Korea. Sci. Rep..

[bib0122] Zomot E., Yardeni E.H., Vargiu A.V., Tam H.-K., Malloci G., Ramaswamy V.K., Perach M., Ruggerone P., Pos K.M., Bibi E. (2018). A new critical conformational determinant of multidrug efflux by an MFS transporter. J. Mol. Biol..

